# Cured Symptoms

**Published:** 1896-08

**Authors:** C. M. Boger

**Affiliations:** Parkersburg, W. Va.


### CURED SYMPTOMS.

C. M. BOGER, M. D., PARKERSBURG, W. VA.

1. Tongue burns like pepper (Iris. Hydras. Xanthy.), headache as if the top of head would fly off. Sense of soreness in stomach. ℞ Iris-v.$^{mm}$ cured.

2. Every time the patient has a cold there is a dripping of clear watery fluid from the uvula (Cep. Merc.-cor. Spig.). ℞ Ceph.$^{m}$ cured.

3. Vertigo, felt most in temples; sensation as if to fall forward; worse from *least motion of the head;* numbness of the extremities; has always been supersensitive to allopathic doses of Morphia. ℞ Morph.-sul.$^{cm}$ cured.

4. Sensation of heaviness in mammæ; fatty pellicle in urine; bloated all over. ℞ Iodum$^{3x}$ cured.

5. Sensation of a lump in the urethra. ℞ Bovista$^{cm}$ cured.

6. Sweat on tips of fingers, excoriating, causing itching and burning in summer. ℞ Sul.$^{cm}$ cured.

7. Awakens and don't know where she is, with great restlessness and diarrhœa. ℞ Ars.$^{30}$ cured.

8. Saliva tastes sweet and is tenacious; aggravated by empty swallowing. Follicular tonsillitis. ℞ Lach.$^{cm}$ cured.

